# Mycophenolate mofetil for systemic sclerosis: drug exposure exhibits considerable inter-individual variation—a prospective, observational study

**DOI:** 10.1186/s13075-020-02323-8

**Published:** 2020-10-06

**Authors:** Kristofer Andréasson, Karl Neringer, Dirk M. Wuttge, Dan Henrohn, Jan Marsal, Roger Hesselstrand

**Affiliations:** 1 Lund University, Skane University Hospital, Department of Clinical Sciences Lund, Rheumatology, Lund, Sweden; 2 Department of Medical Sciences, Uppsala University, Uppsala, Sweden; 3 Lund University, Skane University Hospital, Department of Clinical Sciences Lund, Gastroenterology, Lund, Sweden

**Keywords:** Systemic sclerosis, Scleroderma, Mycophenolate mofetil, Dysbiosis

## Abstract

**Objective:**

Mycophenolate mofetil (MMF) is an established therapy for systemic sclerosis (SSc), but its pharmacokinetics in this disease remains unexplored. We have investigated drug exposure in MMF-treated patients with SSc in relation to clinical features of the disease and common concomitant drugs.

**Methods:**

This study was predefined to include 35 MMF-treated SSc patients who were using MMF at a fixed dose of 0.5, 1.0 or 1.5 g twice daily since at least 3 months. The 12-h drug exposure of the active MMF metabolite mycophenolic acid (MPA) was estimated by repeated analysis of plasma MPA over a 6-h period. This 12-h drug exposure was dose normalised to a daily intake of 3 g MMF (MPA_AUC_3g_) in order to compare subjects using MMF at different doses. Drug exposure was analysed in reference to the clinical characteristics including body weight, renal function, autoantibodies, intestinal dysbiosis, intestinal inflammation assessed by faecal (F)-calprotectin, intestinal symptoms assessed by the University of California Los Angeles Scleroderma Trial Consortium Gastrointestinal Tract Instrument 2.0 and concomitant drug usage including proton-pump inhibitors (PPI).

**Results:**

Thirty-four out of 35 study participants completed the study. The mean daily MMF dose was 2.1 g. Drug exposure expressed as MPA_AUC_3g_ varied up to 8-fold between patients (median 115, range 27–226 mg h/L).

MPA_AUC_3g_ was inversely related to body weight (*r*_*s*_ = − 0.58, *p* < 0.001) and renal function (*r*_*s*_ = − 0.34, *p* = 0.054). Anti-topoisomerase-1 antibodies and male sex were associated with lower MPA_AUC_3g_ (87 vs 123 and 71 vs 141; *p* = 0.008 and *p* = 0.015, respectively). MPA_AUC_3g_ was inversely related to the intestinal abundance of lactobacilli and to F-calprotectin (*r*_*s*_ = − 0.54, *p* = 0.004; *r*_*s*_ = − 0.36, *p* = 0.034), but not to gastrointestinal symptoms. MPA_AUC_3g_ was inversely related to PPI usage (*r*_*s*_ = − 0.45, *p* = 0.007). We found no association between MPA_AUC_3g_ and disease subtype, disease duration or disease activity.

**Conclusion:**

MMF-treated SSc patients exhibit considerable inter-individual variation in drug exposure, and lower MPA levels were primarily found in PPI users with poor prognostic factors. Body weight, renal function, sex, serology, gastrointestinal manifestations and/or measuring individual MPA exposure should be considered when using MMF for SSc.

## Introduction

Mycophenolate mofetil (MMF) was introduced in 1995 as an immunosuppressant to prevent graft rejection in recipients of solid organ transplants [1]. Today, MMF is also an established therapy in systemic sclerosis (SSc) [2–5].

The pharmacokinetics of MMF is complex [6, 7]. Briefly, ingested MMF is hydrolised to the active metabolite mycophenolic acid (MPA) in the stomach and then rapidly absorbed. MPA is converted to the inactive metabolite MPA-7-*O*-glucoronide (MPAG) in the liver and kidney and excreted in the bile and urine. MPAG that reaches the intestine is deconjugated to MPA by anaerobe bacteria and readily reabsorbed [7].

Individual MPA exposure is difficult to estimate based on a single analysis of plasma MPA. A good estimation of MPA exposure can be made by measuring plasma MPA multiple times during a 3–6-h period and calculating MPA area under the concentration-time curve 0–12 h (MPA_AUC_0–12_) [6–8].

The treatment effect of MMF in recipients of solid organ transplants has been studied in relation to MPA_AUC_0–12_. Levels below 30 mg h/L have been associated with increased risk of irreversible transplant failure while exposure above 60 mg h/L did not improve outcomes, but caused more frequent side effects. Consequently, monitoring of MPA exposure has been advocated for recipients of solid organ transplants [6].

The pharmacokinetics of MMF has also been investigated in systemic lupus erythematosus (SLE) and chronic graft versus host disease (GvHD), where data suggests that drug efficacy is dependent on plasma concentration and not on dose [9, 10]. In SLE, the treatment effect levelled out when MPA exposure exceeded 45 mg h/L and a target MPA_AUC_0–12_ level of 35 mg h/L has been suggested for this disease [9]. In GvHD, a drug exposure of at least 30 mg h/L has been suggested [10].

Gastrointestinal involvement in SSc is multifaceted and has been associated with dysbiosis and malabsorption [11, 12]. Dysbiosis in SSc has been characterised by a relative abundance of lactobacilli in three independent studies [12–14]. Malnutrition and malabsorption have been associated with poor outcome in new-onset SSc and altered uptake of the drug penicillamine [15, 16].

In SSc, MMF at a target dose of 3 g daily has shown equivalent efficacy as cyclophosphamide against lung fibrosis [2]. Later studies have confirmed a role for MMF in the treatment of skin and lung fibrosis in SSc, albeit our knowledge on optimal dosing remains limited [3–5, 17]. Both the immunosuppressive and the antifibrotic effects of MPA are concentration-dependent [6, 18].

The purpose of this study was to investigate MPA exposure in SSc subjects treated with MMF. Our secondary objective was to investigate how the pharmacokinetics of MMF relates to clinical characteristics including gastrointestinal factors and to medications commonly used in SSc.

## Patients and methods

### Patients

This prospective open-label study encompassed patients with SSc fulfilling the 2013 ACR/EULAR classification criteria who were using MMF tablets in a fixed dose twice daily since ≥ 3 months [19]. Participants were recruited from all over Sweden with the help of the national patient organisation for SSc. Based on our current knowledge on the pharmacokinetics of MMF, patients with a history of gastrointestinal surgery including resection of any part of the gastrointestinal tract, renal failure or pulmonary arterial hypertension were excluded [7, 20]. Because of the necessity of multiple venepunctures, we excluded patients with a history of complicated venepunctures or anaemia. Further exclusion criteria included pregnancy, ongoing infection and previous solid organ transplantation.

### Patient and public involvement

The study design was developed in collaboration with the national patient organisation for SSc.

### Mycophenolate mofetil

Participants spent the night at the hospital or in direct adjunction to the hospital. They were instructed to follow their usual morning routine regarding concomitant intake of food and other medications together with MMF. Blood samples were drawn right before ingestion of the morning dose (which was administered under supervision) and 60, 180, and 360 min later. Plasma MPA levels were measured using high-performance liquid chromatography. MPA exposure was estimated based on a model previously developed for autoimmune diseases: MPA_AUC_0–12_ = 12.3 + 4.7 × *C*_0_ + 1.2 × *C*_1_ + 2.7 × *C*_3_ + 1.8 × *C*_6_ mg h/L [8].

### Patient characteristics

Disease subtype, antibody status, disease duration since first non-Raynaud’s manifestation, disease activity and skin involvement were defined in accordance with the EUSTAR recommendations [21]. Renal function was estimated based on a combination of measurement of plasma creatinine and plasma cystatin C [22]. Gastrointestinal microbiota dysbiosis was analysed with the GA-map™ Dysbiosis Test (Genetic Analysis, Oslo, Norway) and the relative abundance of lactobacilli analysed [12]. Gastrointestinal inflammation was assessed by faecal (F)-calprotectin (Calpro AS, Norway), and levels above 50 μg/g were considered pathological [11]. Gastrointestinal symptoms were evaluated by the University of California Clinical Trial Consortium Gastrointestinal Tract Instrument (UCLA SCTC GIT) 2.0 [23]. Disease activity was assessed by the EULAR revised activity index [24] and malnutrition risk by the Malnutrition Universal Screening Tool (MUST) [25]. Participants were asked to report recent intake of proton-pump inhibitors (PPI), calcium channel blockers and NSAID by filling out designated forms.

### Statistical analyses

The study was predefined to include thirty-five patients, a number that was chosen together with a senior statistician to be able to do subgroup analyses [6]. In order to compare the relative drug exposure in patients prescribed different doses of MMF, we constructed the variable MPA_AUC_3g_ corresponding to a daily intake of 3 g MMF. This estimate was made by multiplying MPA_AUC_0–12_ by 3 or 1.5, respectively, in patients using MMF at a dose of 1 or 2 g daily [26, 27]. Descriptive data were presented using mean ± (SD) or median (IQR or range), and comparative analyses were done using non-parametric statistics including Spearman’s rank correlation (*r*_*s*_) coefficient, Kruskal-Wallis test and the Mann-Whitney *U* test.

Based on our current knowledge on the pharmacokinetics of MMF, we studied MPA exposure in relation to weight, renal function and concomitant medications [6, 28].

Based on previous reports on malabsorption in SSc, we also set out to explore MPA exposure in relation to gastrointestinal symptoms and inflammation, the MUST and intestinal microbiota [11–16, 23, 25].

Based on current knowledge on SSc prognosis for specific SSc subset, we also set out to explore MPA exposure in relation to skin involvement and serological profile [29].

### Ethics

This study was approved by the Swedish Ethical Review Authority (Dnr 2018/490) and the Swedish Medical Products Agency (EudraCT 2018-002105-54) and prospectively registered at ClinicalTrials.gov (NCT03678987, posted September 20, 2018). All participants gave their written informed consent prior to entering the study. The study was conducted in accordance with the Declaration of Helsinki.

## Results

Thirty-four out of predefined 35 study participants completed the study. Patient characteristics are presented in Table 1 and show some heterogeneity with regard to the MMF dose. Most notably, renal function that was lower in patients using MMF at a lower dose.

**Table 1:** Patient characteristics. Data are shown as numbers and per cent (%) or means ± standard deviation (SD) or median interquartile range (IQR)/range and in relation to daily dose MMF

	***N*** **(%)**	**Daily dose MMF (*n*)**		
		**1 g (5)**	**2 g (21)**	**3 g (8)**
Disease subtype (dcSSc/lcSSc)	12/22 (35/65)	0/5	9/12	3/5
Sex (females/males)	30/4 (88/12)	5/0	19/2	6/2
ANA positivity, of which	33 (97)	5	20	8
ATA	8 (24)	1	5	2
ARA	4 (12)	0	4	0
PM-SCL (75 or 100)	10 (29)	3	4	3
MMF producer (Roche/Actavis/Sandoz)	8/24/2 (24/71/6)	1/4/0	4/16/1	3/4/1
MMF indication				
*Lung fibrosis*	21 (62)	4	10	7
*Skin fibrosis*	8 (24)		8	0
*Combination of disease manifestations*	5 (15)	1	3	1
Dysbiosis Index (1–5) (*n* = 27)	13/14 (48/52)	1/2	9/7	3/5
PPI (daily, sporadic, none)	26/2/6 (76/6/18)	5/0/0	16/1/4	5/1/2
CCB (daily, sporadic, none)	26/0/8 (76/0/24)	3/0/2	17/0/4	6/0/2
NSAID (daily, sporadic, none)	2/1/31 (6/3/91)	1/0/4	1/0/20	0/1/7
MUST (0, ≥ 1)	30/4 (88/12)	5/0	3/18	1/8
F-calprotectin pathological (yes/no)	14/20 (41/59)	3/2	9/12	2/6
	**Mean ± SD**	**Mean ± SD**	**Mean ± SD**	**Mean ± SD**
Age (years)	58 (15)	62 (14)	59 (15)	51 (16)
Weight (kg)	73 (16)	70 (15)	71 (14)	80 (23)
EUSTAR Revised Activity Index	2.30 (1.48)	3.0 (1.8)	2.2 (1.6)	2.1 (1.1)
Leukocyte count (10^9^/L)	7.3 (2.6)	8.4 (4.2)	7.2 (2.3)	6.6 (2.3)
Lymphocyte count(10^9^/L)	1.4 (0.6)	1.7 (1.0)	1.4 (0.47)	1.7 (0.83)
Albumin (g/L)	42 (3.5)	40 (3.7)	42 (3.6)	44 (2.9)
eGFR (ml/min/1.73 m^2^)	76 (12)	66 (6.1)	76 (12)	83 (9.0)
	**Median (IQR)**	**Median (range)**	**Median (range)**	**Median (range)**
Disease duration (years)*	4.9 (2.7–8.1)	4.8 (1.4–25)	5.8 (1.0–17)	3.7 (0.3–6.1)
Duration since SSc diagnosis (years)	3.5 (1.2–7.1)	3.6 (1.0–9.8)	4.3 (0.0–18)	1.2 (0.7–10)
Duration of MMF treatment (years)	2.1 (1.1–4.3)	2.5 (0.8–9.5)	2.6 (0.3–6.9)	0.9 (0.5–9.3)
Faecal calprotectin	39 (12–102)	74 (32–194)	41 (4–443)	17 (1–201)
UCLA SCTC GIT 2.0 Total score (*n* = 31)	0.24 (0.04–0.66)	0.125 (0.0–1.2)	0.31 (0.0–1.5)	0.25 (0.0–1.2)

*dcSSc* diffuse cutaneous systemic sclerosis, *lcSSc* limited cutaneous systemic sclerosis, *ATA* anti-topoisomerase-1 antibodies, *ARA* anti-RNA-polymerase III antibodies, *PM-SCL* anti-polymyositis-scleroderma, *MMF* mycophenolate mofetil, *PPI* proton-pump inhibitor, *SD* standard deviation, *UCLA SCTC GIT 2.0* University of California Los Angeles Scleroderma Clinical Trials Consortium Gastrointestinal Tract Instrument 2.0, *eGFR* estimated glomerular filtration rate

*Since first non-Raynaud’s manifestation

The mean daily MMF dose was 2.1 g. MPA exposure exhibited considerable variation between patients (Table 2). Two subjects exhibited an estimated MPA_AUC_0–12_ < 30 mg h/L while 25 subjects exhibited an estimated MPA_AUC_0–12_ > 60 mg h/L. The MPA_AUC_0–12_ displayed a linear dose-dependent relationship with MMF intake (Table 2), and MPA_AUC_3g_ was therefore used for further analyses.

**Table 2:** MPA exposure in MMF-treated systemic sclerosis. The mean and median MPA exposure correlated to MMF intake in a dose-dependent manner. There was a considerable inter-individual variation in MPA exposure in all three subgroups

Daily dose MMF	MPA_AUC_**0–12**_ (mg h/L)				MPA_AUC_**3g**_ (mg h/L) (***n*** = 34)
	0.5 g × 2 (***n*** = 5)	1 g × 2 (***n*** = 21)	1.5 g × 2 (***n*** = 8)	All subjects (***n*** = 34)	
Mean	50	75	102	78	115
Median	48	72	119	72	114
Interquartile range	33–69	60–86	72–135	58–102	87–139
Range	25–75	43–120	27–139	25–139	27–226

*MPA_AUC*_*0–1*2_ mycophenolate acid area under the concentration-time curve 0–12 h (mg h/L), *MMF* mycophenolate mofetil

*MPA_AUC*_*3g*_ MPA_AUC_0–12_ adjusted to a daily intake of 1.5 g MMF twice daily (mg h/L)

MPA_AUC_3g_ varied inversely with body weight (*r*_*s*_ = − 0.58, *p* < 0.001) and the estimated glomerular filtration rate (*r*_*s*_ = − 0.34, *p* = 0.054). Male subjects had lower MPA_AUC_3g_ levels than their female counterparts (74 vs 121 mg h/L; *p* = 0.015; Supplemental Figure 1). Of note, the correlation between MPA_AUC_3g_ and renal function and sex could not be corroborated when relating these variables to MPA exposure expressed as MPA_AUC0–12 (Table 3).

**Table 3:** Associations between MPA exposure and clinical characteristics

MPA exposure estimate, daily dose MMF	All patients (***N*** = 34)		MPA_AUC_**0–12**_, mg h/L, median (range)		
	MPA_AUC_**3g**_, mg h/L, median (IQR)	MPA_AUC_**0–12**_, mg h/L, median (IQR)	1 g × 2 (***n*** = 21)	1 g × 2 (***n*** = 21)	1.5 g × 2 (***n*** = 8)
Disease subtype (lcSSc vs dcSSc)	119 (90–132) vs 98 (85–150), *p* = 0.929	72 (48–85) vs 75 (59–116), *p* = 0.383	N/A	73 (43–101) vs 65 (56–121)	118 (27–139) vs 125 (83–139)
Sex (male vs female)	74 (36–103) vs 121 (94–141), *p* = 0.015	58 (31–81) vs 72 (60–103), *p* = 0.262	N/A	58 (43–73) vs 71 (47–121)	55 (27–83) vs 123 (68–139)
Topoisomerase (positive vs rest)	87 (67–108) vs 123 (97–146), *p* = 0.008	58 (31–71) vs 78 (63–103), *p* = 0.039	25 (25) vs 56 (41–75)	61 (43–72) vs 77 (47–121)	72 (27–118) vs 123 (68–139)
F-calprotectin (pathological vs normal)	99 (74–119) vs 127 (100–144), *p* = 0.025	57 (42–71) vs 83 (69–116), *p* = 0.001	41 (25–63) vs 62 (48–75)	58 (43–101) vs 82 (61–121)	72 (27–118) vs 123 (68–139)
Dysbiosis index ≥ 3	114 (74–139) vs 120 (86–141), *p* = 0.756	71 (57–119) vs 81 (61–94), *p* = 0.943	44 (25–63) vs 75	65 (43–104) vs 81 (56–121)	125 (68–139) vs 83 (27–120)
MUST pathological	108 (87–134) vs 114 (87–141), *p* = 0.817	72 (58–124) vs 72 (58–102), *p* = 0.857	N/A	N/A	N/A
PPI (user vs non-user)	108 (84–137) vs 121 (108–143), *p* = 0.341	67 (56–86) vs 92 (72–125), *p* = 0.060	N/A	66 (43–120) vs 76 (31–103)	100 (27–139) vs 130 (120–139)
CCB (user vs non-user)	109 (87–141) vs 122 (87–135), *p* = 0.765	73 (60–102) vs 63 (49–115) *p* = 0.591	48 (25–75) vs 52 (41–63)	72 (43–121) vs 59 (47–83)	100 (27–139) vs 132 (125–139)
NSAID (user vs non-user)	120 vs 108 (84–139) *p* = 0.524	73 vs 72 (58–101) *p* = 0.909	N/A	N/A	N/A
**Spearman’s rank correlation coefficient**					
Body weight	*r*_*s*_ = − 0.58, *p* < 0.001	*r*_*s*_ = − 0.38, *p* = 0.029	*r*_*s*_ = − 0.50	*r*_*s*_ = − 0.55	*r*_*s*_ = − 0.48
Age	*r*_*s*_ = 0.33, *p* = 0.053	*r*_*s*_ = 0.10, *p* = 0.574	*r*_*s*_ = 0.90	*r*_*s*_ = 0.32	*r*_*s*_ = − 0.24
Disease duration	*r*_*s*_ = − 0.10, *p* = 0.566	*r*_*s*_ = − 0.244, *p* = 0.164	*r*_*s*_ = − 0.30	*r*_*s*_ = − 0.21	*r*_*s*_ = 0.29
Years on MMF	*r*_*s*_ = 0.29, *p* = 0.102	*r*_*s*_ = 0.52, *p* = 0.002	*r*_*s*_ = 0.50	*r*_*s*_ = 0.18	*r*_*s*_ = 0.53
Disease activity	*r*_*s*_ = − 0.06, *p* = 0.728	*r*_*s*_ = − 0.17, *p* = 0.349	*r*_*s*_ = − 0.50	*r*_*s*_ = 0.08	*r*_*s*_ = − 0.667
eGFR	*r*_*s*_ = − 0.34, *p* = 0.054	*r*_*s*_ = − 0.024, *p* = 0.894	*rs* = − 0.30	*rs* = − 0.21	*rs* = − 0.56
Albumin	*r*_*s*_ = − 0.17, *p* = 0.351	*r*_*s*_ = 0.06, *p* = 0.762	*r*_*s*_ = 0.95	*r*_*s*_ = − 0.34	*r*_*s*_ = − 0.22
PPI dose	*r*_*s*_ = − 0.45, *p* = 0.007	*r*_*s*_ = − 0.43, *p* = 0.012	*r*_*s*_ = − 0.71	*r*_*s*_ = − 0.52	*r*_*s*_ = − 0.31
UCLA GIT 2.0	*r*_*s*_ = 0.16, *p* = 0.358	*r*_*s*_ = 0.15, *p* = 0.385	*r*_*s*_ = 0.90	*r*_*s*_ = 0.159	*r*_*s*_ = − 0.048
F-calprotectin	*r*_*s*_ = − 0.36, *p* = 0.034	*r*_*s*_ = − 0.60, *p* = 0.010	*r*_*s*_ = − 0.31	*r*_*s*_ = − 0.53	*r*_*s*_ = − 0.50
Lactobacilli	*r*_*s*_ = − 0.54, *p* = 0.004	*r*_*s*_ = − 0.49, *p* = 0.010	N/A	*r*_*s*_ = − 0.60	*r*_*s*_ = − 0.14
**Kruskal-Wallis one-way analysis of variance**					
MMF indication	*p* = 0.699	*p* = 0.513	N/A	N/A	N/A
Serology	*p* = 0.005	*p* = 0.297	N/A	N/A	N/A
MMF brand	*p* = 0.897	*p* = 0.746	N/A	*p* = 0.512	*p* = 0.547

*MPA_AUC*_*0–12*_ mycophenolate acid area under the concentration-time curve 0–12 h (mg h/L), *MPA_AUC*_*3g*_ MPA_AUC_0–12_ adjusted to a daily intake of 1.5 g MMF twice daily (mg h/L), *MMF* mycophenolate mofetil, *dcSSc* diffuse cutaneous systemic sclerosis, *lcSSc* limited cutaneous systemic sclerosis, *MUST* Malnutrition Universal Screening Tool [25], *NSAID* non-steroidal anti-inflammatory drug, *CCB* calcium channel blocker, *PPI* proton-pump inhibitor, *eGFR* estimated glomerular filtration rate, *UCLA GIT 2.0* University of California Los Angeles Scleroderma Trial Consortium Gastrointestinal Tract Instrument 2.0 [23]

**Fig. 1:**
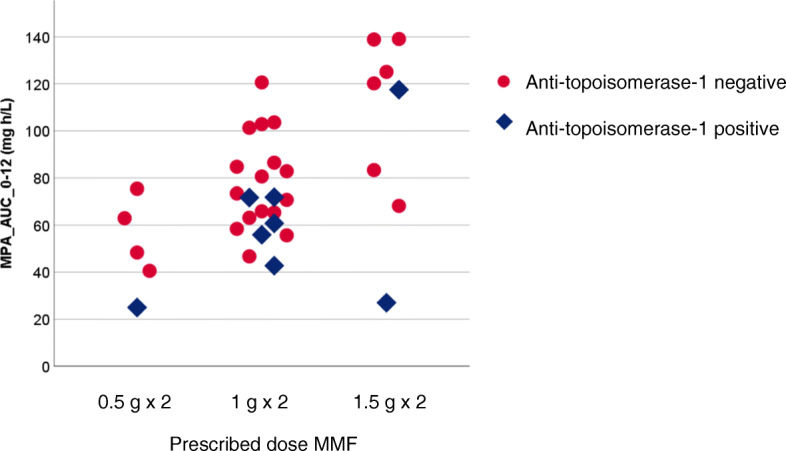
MPA exposure in relation to daily MMF intake. MPA exposure, defined by the variable MPA_AUC_0–12_ varied considerably between patients. Patients with anti-topoisomerase-1 antibodies had significantly lower drug exposure compared to the other subjects

MPA exposure exhibited significant heterogeneity in relation to four serological groups (Table 3, *p* value for between-group differences = 0.005). Patients with anti-topoisomerase-1 autoantibodies had lower MPA_AUC_3g_ compared to other participants (median 87 vs 123 mg h/L; *p* = 0.008; Table 3, Fig. 1).

**Fig. 2:**
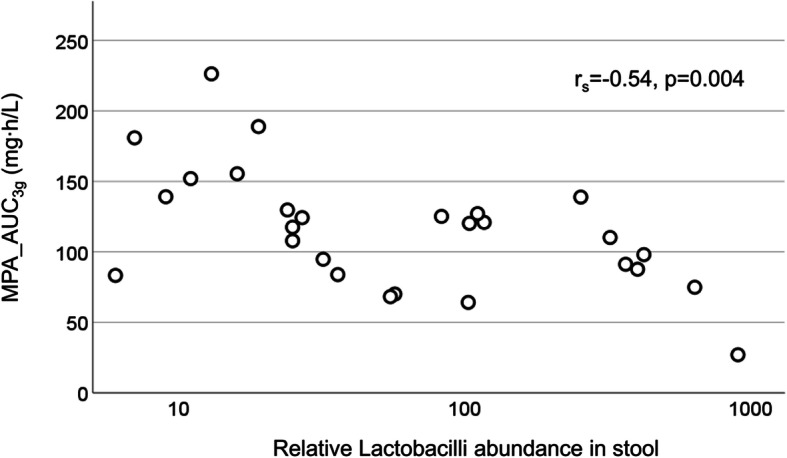
MPA exposure in relation to intestinal lactobacilli levels. Patients with increased levels of lactobacilli in their stool had lower MPA exposure

Dysbiosis was present in 14 of the 27 patients tested and was not associated with altered MPA exposure (Table 3). However, there was a negative association between the relative prevalence of lactobacilli and MPA_AUC_3g_ (*r*_*s*_ = 0.54, *p* = 0.004; Fig. 2). Patients with normal F-calprotectin had higher MPA_AUC_3g_ compared to patients with pathological F-calprotectin levels (127 vs 99 mg h/L, *p* = 0.040), and F-calprotectin levels correlated inversely with MPA_AUC_3g_ (*r*_*s*_ = − 0.36, *p* = 0.025). We were unable to find an association between MPA exposure and gastrointestinal symptoms or malnutrition assessed by the UCLA SCTC GIT 2.0 and MUST (Table 3). However, MPA_AUC_3g_ was negatively associated with PPI usage (*r*_*s*_ = − 0.45, *p* = 0.007; Supplemental Figure 1).

We were not able to find any association between MPA exposure and calcium channel blockers or NSAID usage, nor disease duration, diffuse cutaneous disease subtype or disease activity (Table 3).

All five patients prescribed MMF at a dose of 1 g daily had previously used the drug at a higher dose. Of these, three had experienced suspect adverse events resulting in lowering of the dose while two patients were prescribed the lower dose of 1 g twice daily when their disease was considered stable. In total, five patients reported adverse events associated with MMF. A history of adverse events was not associated with MPA_ AUC_3g_ (*p* = 0.508).

## Discussion

MMF is an immunosuppressive and anti-fibrotic drug that shows a plasma concentration-dependent efficacy when used for solid organ transplantation, SLE and GvHD [1, 9, 10]. It is an established therapy in SSc where available data support its use at a target dose of 3 g daily [2]. We report considerable inter-individual variation in MPA exposure following oral MMF treatment in SSc. We also report that 25 out of 34 of the study participants exhibited MPA_AUC_0–12_ above the recommended interval for recipients of solid organ transplantation (30–60 mg h/L) [6], even though the average daily dose was only 2.1 g. These results need to be validated but indicate that it is common that SSc patients treated according to the current guidelines are exposed to higher levels of MPA than has been recommended for other diseases. One should be careful not to extrapolate safety data from previous MMF studies, to SSc patients prescribed MMF at a daily dose of 2 or 3 g.

In this study, we found that anti-topoisomerase-1 positivity and male sex predicted lower level of MPA exposure. We also identified that both an SSc-specific alteration in the microbiota and gastrointestinal inflammation were associated with lower levels of MPA exposure, suggesting that gastrointestinal manifestations of SSc may alter the bioavailability of MPA. We do not know what drug exposure is optimal for SSc. Our findings could be of clinical importance for newly diagnosed anti-topoisomerase-1 positive male patients with malabsorption. These patients have a poor prognosis, partly because of progressive lung fibrosis, and are in need of the full therapeutic potential of MMF [15, 29].

We found that PPI intake was associated with lower MPA exposure which is in line with previous reports. The mechanism behind this finding has been suggested to be explained the pH-dependent hydrolisation of MMF to MPA in the stomach [30]. This finding is of clinical significance considering the frequency by which PPIs are used for SSc [5].

Patients with the highest drug exposure had poor renal function and/or low body weight. This finding is in line with previous studies in other diseases and questions the rationale of a fixed target dose for all SSc patients.

The limitations of this study include the fact that we estimated MPA exposure using a 4-point model that has been designed for autoimmune diseases but not validated for SSc or for MPA measurement using high-performance liquid chromatography [8]. This study is limited to 34 patients which must be taken into consideration when extrapolating data from our subgroup analyses. Also, this study includes patients using MMF at three different daily doses with some heterogeneity between the groups, e.g. renal function, age, weight, disease duration and years on MMF (Table 1). Furthermore, we have not investigated the impact of MPA exposure on drug toxicity and drug efficacy. This issue needs further exploration before we can recommend therapeutic drug monitoring in MMF-treated SSc on a routine basis. Finally, because some of the patients that were excluded from the study were likely to suffer from a more severe disease (e.g. patients with a history of complicated venepunctures, renal failure or anaemia), the results may not be representative for the whole SSc population. There may also exist a selection bias towards participants who were able to tolerate this medication.

The optimal therapeutic interval for MPA in SSc remains to be defined. Considering the multiple modes of action by which this drug may modulate autoimmune fibrotic disease [31], it is possible that higher plasma concentrations compared to other diseases are needed in order for the drug to exhibit its full effect in SSc. In vitro studies have shown that not only the immunosuppressive function but also the anti-fibrotic effect of MPA is concentration-dependent [18]. Future studies are needed to investigate the clinical outcome of MMF therapy in SSc in relation to MPA_AUC_0–12_, as has been done in SLE [9]. We therefore advocate MPA exposure to be analysed in clinical SSc trials as was alluded to already by the authors of the SLS-II trial: “while serum levels of MMF [sic] might have provided some insight, they were not obtained” [2].

Individual therapeutic monitoring of MMF together with an appropriate dose adjustment has successfully been used for recipients of solid organ transplants because of the concentration-dependent efficacy of this drug [6]. Based on these experiences, measurement of plasma MPA and estimation of MPA_AUC_0–12_ are currently available in many clinics worldwide.

In the era of personalised medicine, we suggest that individual MPA exposure and its relation to body weight, renal function, prognostic markers and PPI usage should be considered when using MMF in SSc.

## Conclusions

MMF-treated SSc patients exhibit considerable inter-individual variation in drug exposure. Higher levels are found in female patients with poor renal function and low body weight. Relatively lower levels are found in overweight PPI users with poor prognostic factors. Body weight, renal function, sex, serology, gastrointestinal manifestations and the possibility of individual analysis of MPA levels should be considered when using and evaluating MMF for SSc.

## Supplementary information

**Additional file 1:** **Supplemental Figure 1A-E.** Scatter plot of MPA_AUC_3g_ in relation to sex, body weight, renal function, relative omeprazole dose and F-calprotectin.

## Data Availability

The dataset supporting the conclusions of this article is available upon reasonable request.

## Abbreviations

ACR: American College of Rheumatology
EudraCT: European Union Drug Regulating Authorities Clinical Trials Database
EULAR: European League Against Rheumatism
EUSTAR: The European Scleroderma Trials and Research group
GvHD: Graft versus host disease
MMF: Mycophenolate mofetil
MPA: mycophenolic acid
MPA_AUC_0–12_: MPA area under the concentration-time curve 0–12 h
MPA_AUC_3g_: MPA_AUC_0–12_ adjusted to a daily intake of 1.5 g MMF twice daily
MPAG: MPA-7-*O*-glucoronide
MUST: Malnutrition Universal Screening Tool
PPI: Proton-pump inhibitor
SLE: Systemic lupus erythematosus
SSc: Systemic sclerosis
UCLA SCTC GIT: University of California Clinical Trial Consortium Gastrointestinal Tract Instrument
