# Supplementary material for: Mycophenolate mofetil for systemic sclerosis: drug exposure exhibits considerable inter-individual variation—a prospective, observational study
Source: Arthritis Res Ther. 2020 Oct 6;22:230. doi: 10.1186/s13075-020-02323-8 (PMC7539387; doi:10.1186/s13075-020-02323-8)
Supplement: Supplementary file 1 — Additional file 1: Supplemental Figure 1A-E. Scatter plot of MPA_AUC3g in relation to sex, body weight, renal function, relative omeprazole dose and F-calprotectin. [file 13075_2020_2323_MOESM1_ESM.pptx]

## Slide 1
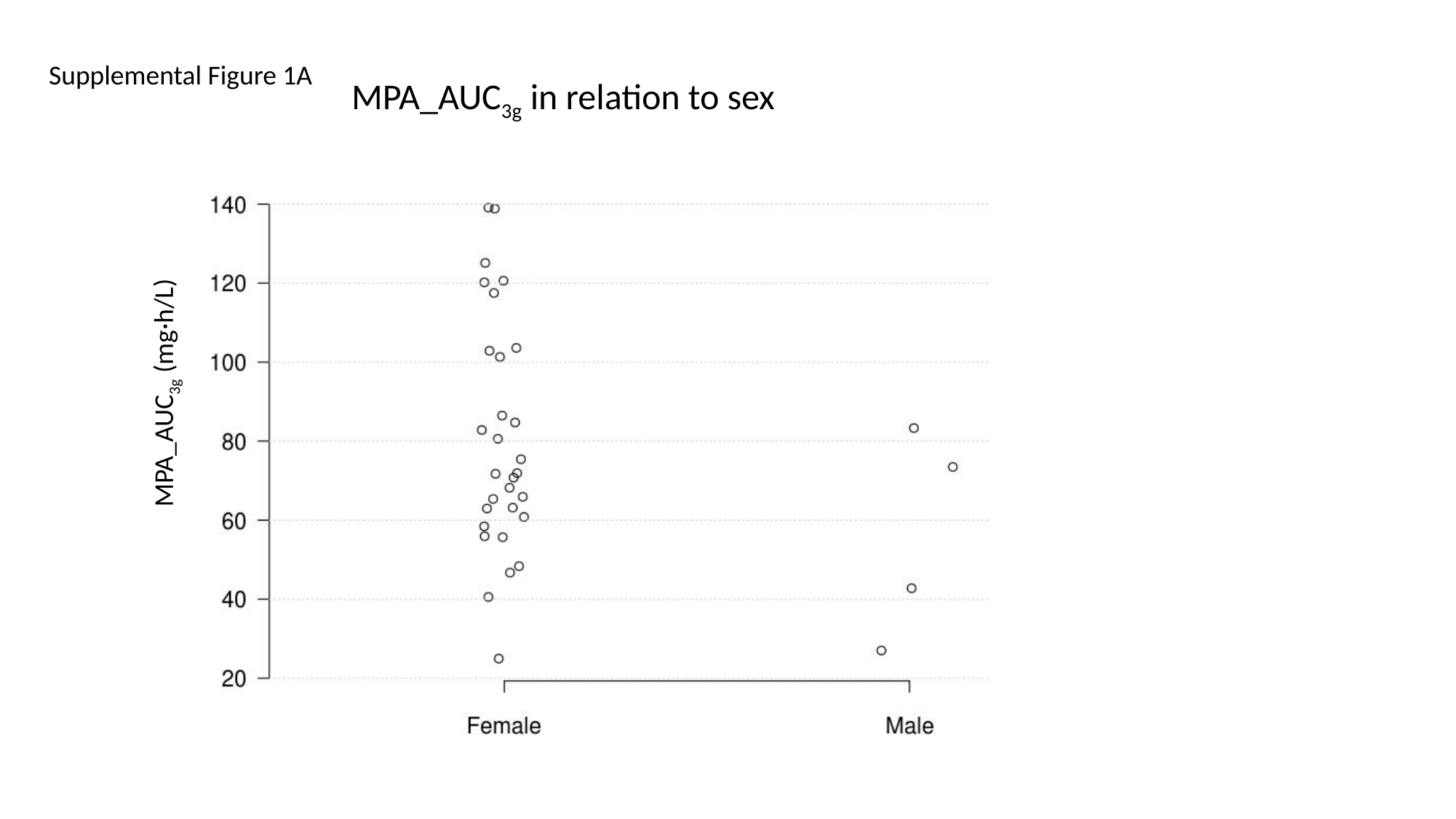

Supplemental Figure 1A
MPA_AUC3g in relation to sex
MPA_AUC3g (mg·h/L)

## Slide 2
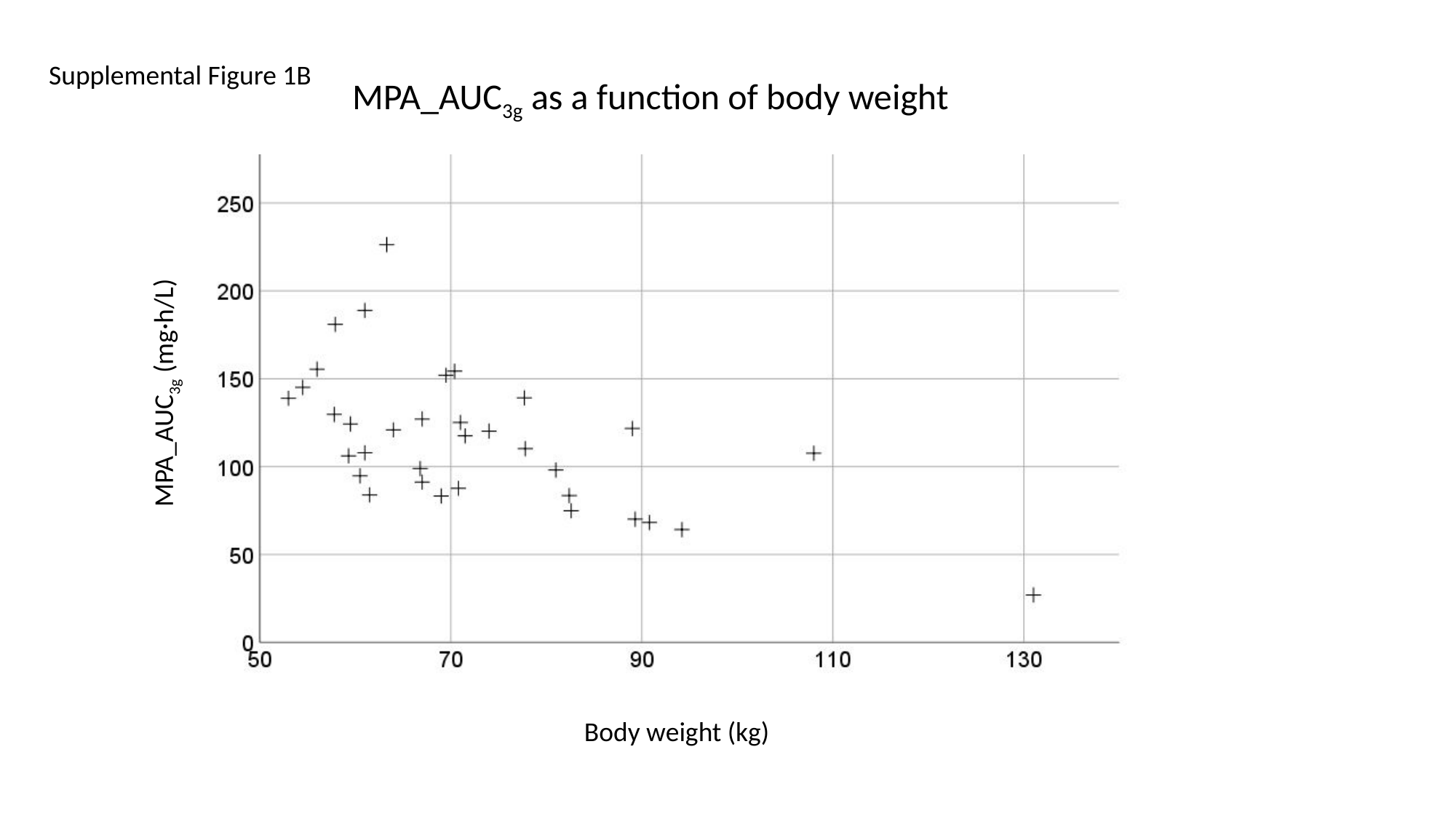

Supplemental Figure 1B
MPA_AUC3g as a function of body weight
MPA_AUC3g (mg·h/L)
Body weight (kg)

## Slide 3
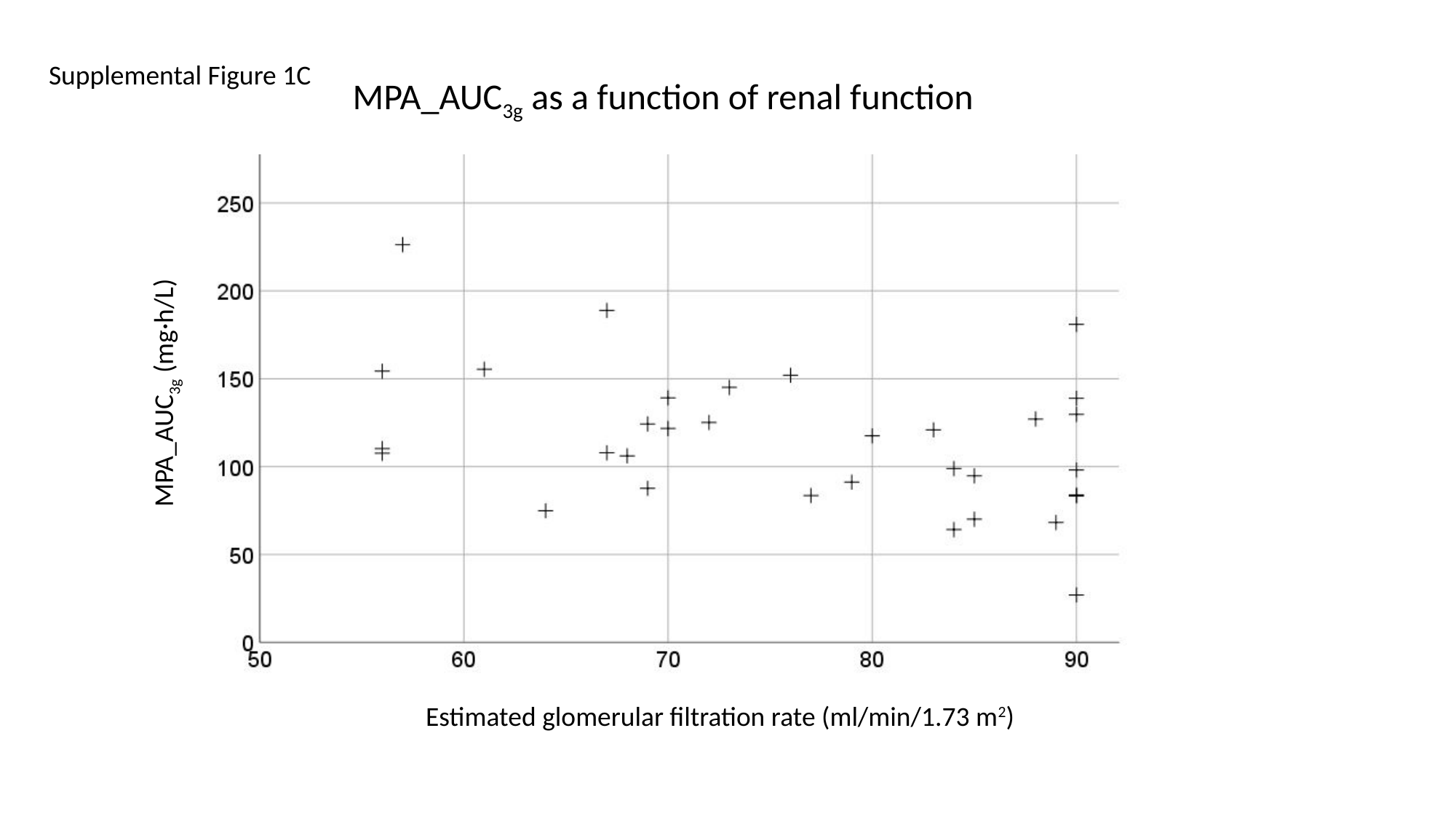

Supplemental Figure 1C
MPA_AUC3g as a function of renal function
MPA_AUC3g (mg·h/L)
Estimated glomerular filtration rate (ml/min/1.73 m2)

## Slide 4
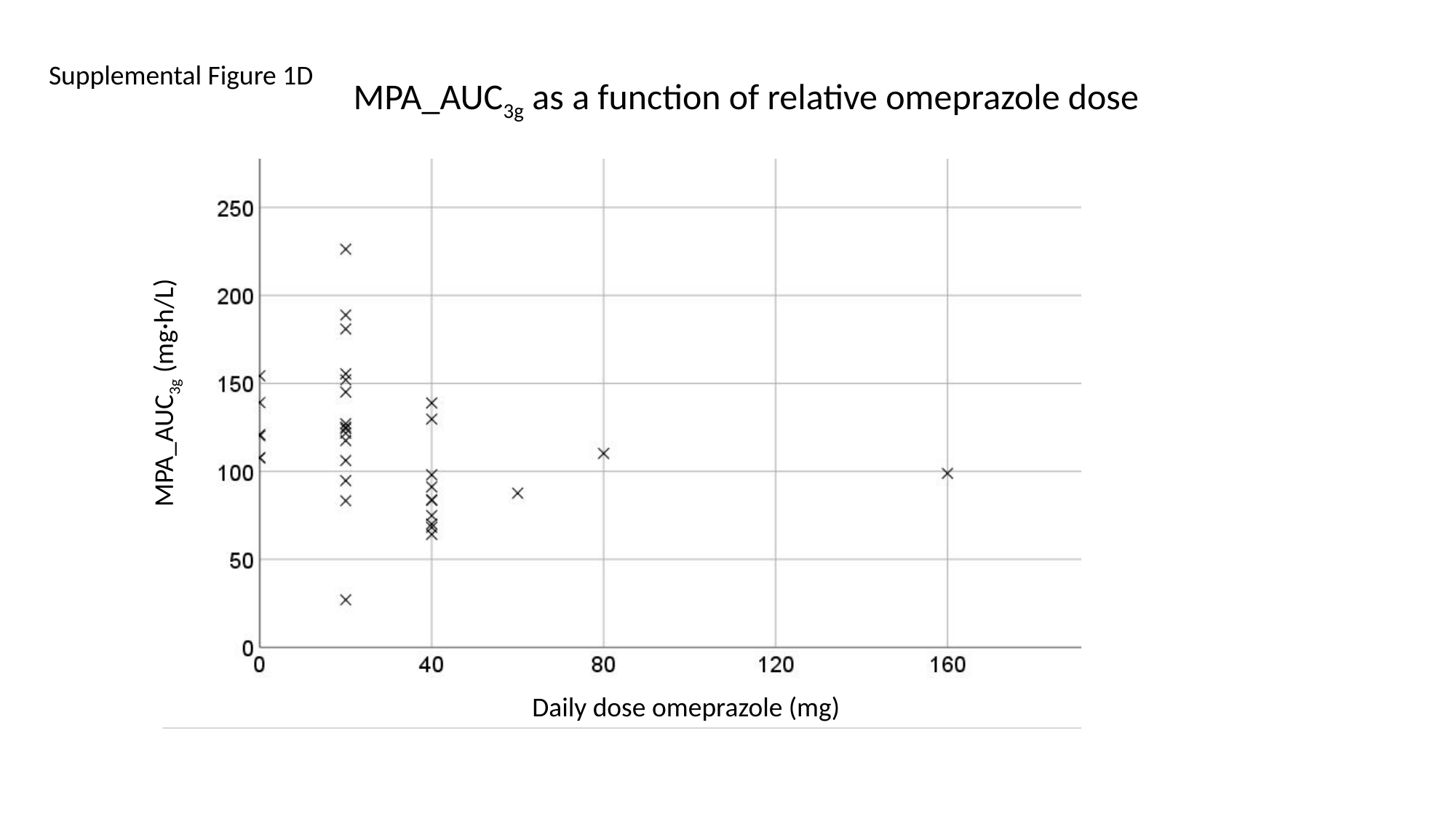

Supplemental Figure 1D
MPA_AUC3g as a function of relative omeprazole dose
MPA_AUC3g (mg·h/L)
Daily dose omeprazole (mg)

## Slide 5
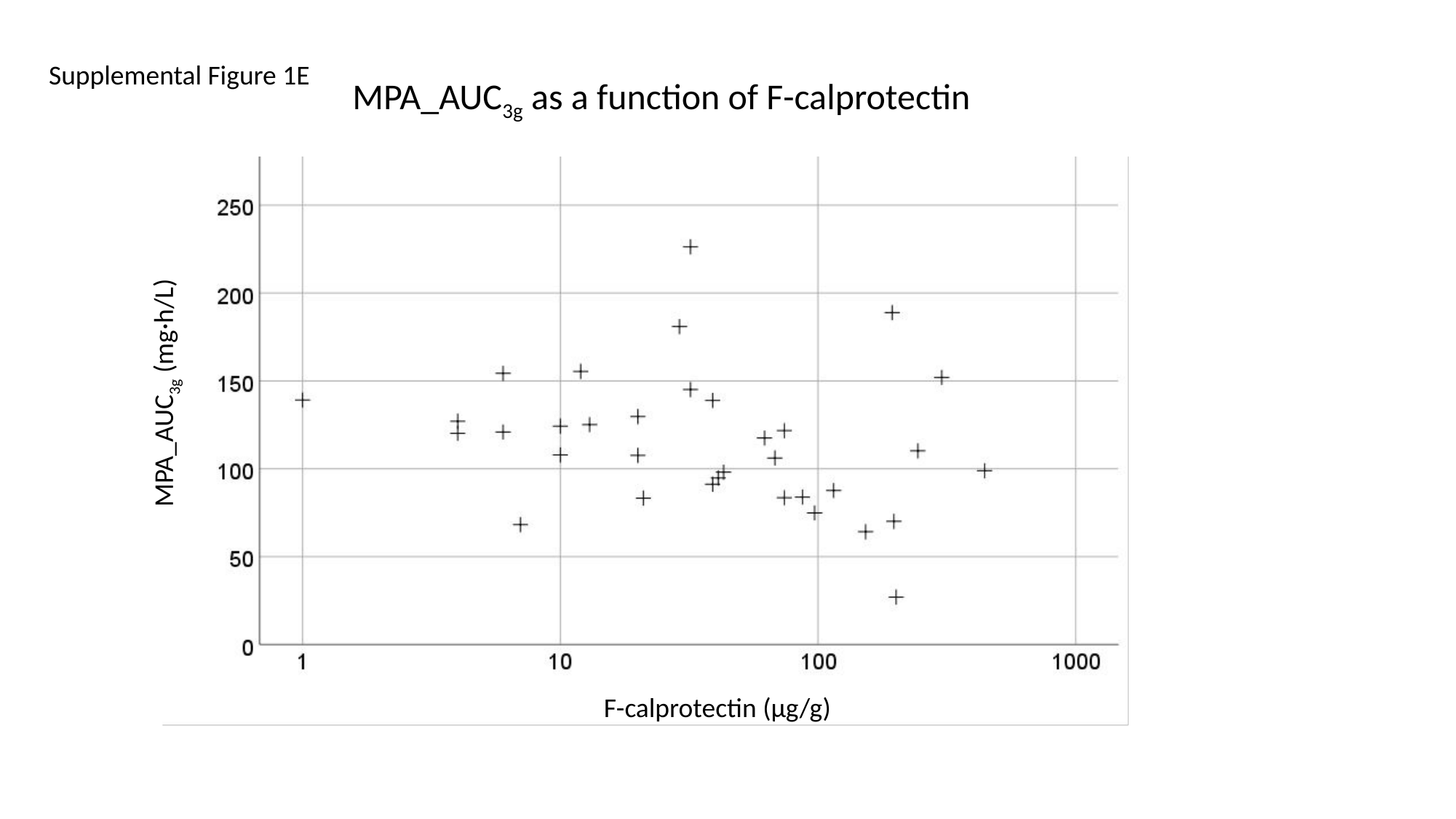

Supplemental Figure 1E
MPA_AUC3g as a function of F-calprotectin
MPA_AUC3g (mg·h/L)
F-calprotectin (µg/g)
